# Comparison of Butorphanol, Methadone, and Pethidine in Combination with Alfaxalone for Premedication in Isoflurane-Anesthetized Cats Undergoing Ovariectomy

**DOI:** 10.3390/ani14131997

**Published:** 2024-07-06

**Authors:** Giulia Moretti, Irene Mattiuzzi, Lisa Garofanini, Eleonora Monti, Benedetta Serni, Antonello Bufalari, Sara Nannarone

**Affiliations:** 1 Department of Veterinary Medicine, University of Perugia, Via San Costanzo 4, 06126 Perugia, Italy; giulia.moretti@unipg.it (G.M.); irene98matt@gmail.com (I.M.); lisa.garofanini@collaboratori.unipg.it (L.G.); eleonoramonti94@gmail.com (E.M.); benedetta.serni@gmail.com (B.S.); antonello.bufalari@unipg.it (A.B.); 2 CeRiDA (Research Center on Animal Pain), Department of Veterinary Medicine, University of Perugia, Via San Costanzo 4, 06126 Perugia, Italy

**Keywords:** acute pain, alfaxalone, behavioral assessment, butorphanol, feline, spaying, methadone, pain score, pethidine

## Abstract

**Simple Summary:**

This study aims to assess the effects of different combinations of opioids (butorphanol, methadone, or pethidine) with alfaxalone as a premedication in cats undergoing elective ovariectomy. Data collected were analyzed to identify an anesthetic protocol that grants a stable surgical anesthetic depth, while preserving cardiorespiratory function and providing adequate analgesia in the perioperative phase. The study results showed good cardiorespiratory stability with pethidine but a low quality of sedation, which is why its use in combination with alfaxalone is not recommended in more feral feline subjects, suggesting also tailoring the choice of the opioid based on the animal’s behavior. The combinations of the injectable anesthetic drugs assessed in this study could be a possible and advantageous choice for short and elective surgical procedures.

**Abstract:**

The aim of this study was to compare three different anesthetic protocols administered intramuscularly (IM) in cats undergoing elective ovariectomy, while evaluating the quality of sedation, antinociceptive, isoflurane-sparing effect, and analgesia in the intra-operative and post-operative phases. A total of 71 female cats were sedated IM with alfaxalone (3 mg/kg) combined with either butorphanol (0.3 mg/kg), methadone (0.3 mg/kg), or pethidine (5 mg/kg). During surgery, vital parameters were constantly monitored; at the end of the procedure, the quality of recovery was assessed through a specific form and each cat was scored for perceived pain using the UNESP-Botucatu scale for 5 days, and rescue analgesia was provided with buprenorphine IM when indicated. Moreover, differences between two different post-operative resting regimens (hospital kennels vs. home) were also assessed. A significant difference emerged for the amount of IM dexmedetomidine required to achieve an adequate level of sedation for intravenous catheterization, highlighting a greater need in the pethidine group (*p* = 0.021). There was no significant difference between opioid groups for the requirement of intra-operative rescue analgesia, and the clinical parameters were kept within physiological ranges regardless of the opioid used in premedication. Lastly, differences between the UNESP-Botucatu scores were detected from day 3 to day 5 post-operatively, with lower scores in cats with home resting regimens compared to the hospitalized animals, likely due to the presence of an unfamiliar condition and the absence of a cat-friendly environment.

## 1. Introduction

Gonadectomy is one of the most common surgical procedures performed in cats in the veterinary practice worldwide. The goal is to reduce overpopulation and stray cats, to prevent diseases of the reproductive tract, and to mitigate the unpleasant behaviors due to hormonal activity [1,2]. At the same time, both ovariectomy and orchiectomy are often used as a clinical model for pain assessment either in dogs or cats [3,4,5]. Nevertheless, pain assessment in cats has been underestimated for years, compared to other species [6,7,8]. This could likely be due to their solitary behavior and their innate tendency to mask the typical signs associated with pain [8,9]; however, awareness of the importance of an adequate analgesic plan and pain assessment in feline patients has recently increased [8,10]. According to the American Association of Shelter Veterinarians’ 2016 guidelines for spay–neuter programs, the selection of anesthetic protocols depends on several factors, i.e., number and type of patients; the skill of the staff, including the competence and speed of the surgeon and anesthetist; and the availability of anesthetic drugs. The key criteria to identify the safest, most humane, fastest, and most convenient anesthetic protocol include analgesics administration, stress reduction, myorelaxation, and unconsciousness [11]. Ideally, a physical examination should be performed before the animal is anesthetized, but anxiety, aggression, or feral behavior may prevent a thorough examination prior to sedation or induction of anesthesia.

Several cost-effective protocols combining injectable and inhalant drugs have been largely described with the goal of obtaining balanced anesthesia and providing high-quality care to optimize patient outcomes [12,13,14]. 

As reported in the guidelines for the recognition, evaluation, and treatment of pain in small animals drawn up by Mathews et al. [15], several options are reported for the management of perioperative pain during gonadectomy in cats. In particular, the recommended protocols include the use of opioids as an analgesic in association with acepromazine or alpha-2-agonists in premedication. Opioids are often the most considered drugs for designing protocols. Although they do not stop the pain signal transmission, they are effective and rapidly acting, making them excellent for acute pain relief. Full μ-opioid receptor agonists (such as methadone or meperidine) are the most potent analgesics, while butorphanol, a μ-antagonist and κ-agonist, is only mildly to moderately potent and has a short duration of action [16].

The combination of alfaxalone with opioids in cats has been largely described [17,18,19,20]. In a previous study conducted by our research group, the injection into the supraspinatus muscle of alfaxalone with either butorphanol, methadone, or pethidine, determined a good quality of sedation for medium–short procedures in cats of any age, ASA status, and BCS [21]. The three opioids were equally safe and effective in providing adequate restraint for procedures lasting about 17 min, with acceptable self-limiting secondary effects. For these reasons, the aim of this study is to compare the same protocols used as a premedication in female cats undergoing elective gonadectomy under isoflurane anesthesia, while assessing anesthetic depth, cardiorespiratory effects, antinociceptive activity, and recovery quality.

## 2. Materials and Methods

The study was approved by the Ethical Committee of the University of Perugia (protocol number: 2019/02) and adhered to internationally recognized high standards (‘best practice’) of individual veterinary clinical patient care [22]. Non-experimental animals were used, and owner consent was obtained for each cat enrolled in the study.

### 2.1. Animals and Protocol

This randomized, investigator-blinded, prospective clinical study included cats without breed restrictions with different living conditions (domestic or stray) admitted from March 2022 to February 2024 to the Veterinary Teaching Hospital for ovariectomy (OV) by ventral midline celiotomy. Final-year intern-students were involved in the procedures, providing hands-on help during both anesthesia and surgery. As is standard for our institution, owners were requested to fast their animal from the night before surgery with water restricted to the early morning of referral (at least 2 h before the procedure). At referral, cats were weighed and, if feasible according to their behavior, they underwent clinical examination. Behavior was assessed through a temperament score that ranged from 1 (very friendly) to 5 (very aggressive) [21]. Clinical examination allowed to define the ASA physical status and body condition score (BCS), and to collect the following physiological parameters: heart rate (HR, beats/min), capillary refill time, respiratory rate (RR, breaths/min), and body temperature (T, °C). No blood tests were performed, neither was ultrasonography used to exclude ongoing pregnancy, but health status was considered based on clinical examination and history when domesticated cats were present. Inclusion criteria included age ≥ 6 months and ASA status of 1 with no restriction on body weight nor breed. Exclusion criteria were a history related to adverse reactions to one or more of the molecules included in the protocol and ongoing pregnancy.

The minimum sample size calculation and post-hoc power analyses were performed using G*Power software (version 3.1.9.6), counting 3 experimental groups (different opioids: methadone, butorphanol, and pethidine) and setting α  =  0.05. Continuous and normally distributed variables were hypothesized. Before data collection, *f*-tests evaluating the effect of the opioid were planned. Setting a power (1 − β)  =  0.95 and an effect size *f*  =  0.82, a minimum of 20 cats per group was required. Thus, after inclusion in the trial, cats were randomly assigned to each group using the function ‘CASUAL’ in Microsoft Excel (version 16.84) until at least 20 cats were included in each group. The achieved power of the analyses, computed using post-hoc procedures and setting the same parameters, was 95%.

According to group allocation, cats would receive an intramuscular (IM) injection in the supraspinatus muscle of one among the three anesthetic protocols: alfaxalone 3 mg/kg (Alfaxan Multidose; Zoetis Italia Srl, Roma, Italy) mixed with either methadone (group M) 0.3 mg/kg (Semfortan, Dechra Veterinary Products Srl, Torino, Italy), butorphanol (group B) 0.3 mg/kg (Dolorex; MSD Animal Health Srl, Milano, Italia), or pethidine (group P) 5 mg/kg (Petidina Cloridrato; Molteni, Scandicci, Italy).

Injections took place in a quiet room with dimmed lighting, and the selected drug mixture was prepared and administered by an anesthetist different from the evaluator. Times to lateral recumbency, quality of induction, and adverse effects such as vomiting, hypersalivation, distress, tremors, myoclonus, and increased muscle tone were recorded [21]. A 2.5 mL syringe with a 22 G needle was used to perform the IM injection, with the cat held in a standing position. The time of injection (T0) was recorded, and the animal was placed back in its transport cage. The reaction to the injection and the degree of the achieved chemical immobilization were scored and recorded as previously described by Giannettoni et al. [21]. An overall sedation score from 1 to 4 (excellent, good, fair, and inadequate) was reported for each cat [21]. If adequate sedation, required to safely place an intravenous catheter, did not occur within 15 min from T0, 3 µg/kg dexmedetomidine (Dextroquillan, A.T.I. S.r.l., Bologna, Italy) would be administered IM, and repeated if no adequate effect appeared within 5 min.

A 22-24 G catheter was aseptically placed in a cephalic vein; thereafter, propofol (Propovet; Zoetis Italia Srl, Roma, Italy) was administered IV to allow for trachea intubation, followed by instillation of 0.5% lidocaine (Lidocaina 2%, Esteve spa, Milano, Italy) drops over the larynx. Anesthesia was maintained with isoflurane (Vetflurane, Virbac Srl, Milano, Italy) in 100% oxygen (250 mL/kg/min) through a non-rebreathing circuit (Mapleson D type). From this time onward, cats were instrumented to record physiological parameters with an electrocardiogram to measure HR, a pulse oximeter probe placed on the tongue for hemoglobin oxygen saturation (SpO_2_, %), and a thermometer advanced into the esophagus to monitor T. Airway gases were continuously sampled and analyzed by a side stream capnograph for the end-tidal partial pressure of carbon dioxide (etCO_2_, mmHg) and end-tidal isoflurane concentration (etIso, %). Blood pressure was measured non-invasively with an oscillometric technique, and the dedicated cuff was placed over a metacarpal/tarsal artery to record the systolic, mean, and diastolic blood pressures (SAP, MAP, DAP). All variables were recorded every 5 min from a multiparametric monitor (Multiparametric monitor-NC12-V, Shenzhen Comen Medical Instruments Co., Ltd., Shenzhen, China). The ventral caudal abdomen was surgically prepared, and before moving into the operating theater, cats received meloxicam (Metacam 0.5%, Boehringer Ingelheim Animal Health Italia Spa, Padova, Italy) 0.1 mg/kg subcutaneously and cefazolin (Cefazolina Teva; Teva Italia Srl, Milano, Italy) 30 mg/kg IV. A ringer lactate solution was administered IV at 2–5 mL/kg/h thorough a peristaltic pump. Animals were placed in dorsal recumbency over a heating device covered with a padded surface and left in spontaneous ventilation unless hypoventilation (RR < 6 breaths/min or etCO_2_ > 50 mmHg) was detected.

### 2.2. Intra-Operative Phase: Monitoring and Maintenance of General Anesthesia

During surgery, vital parameters and etIso were recorded every 5 min and at predefined time-points. These time-points included the following: Backhaus application (BK), start of surgery (SS, considered as skin incision), first ovary search (SO1), traction of first ovary (TO1), eventual first rescue analgesia (R1) and 5 min later (5R1), search for second ovary (SO2), traction of second ovary (TO2), eventual second rescue analgesia (R2), and 5 min later (5R2), start of subcutaneous suture (SQ) end of surgery (ES, considered as the last skin stitch).

For each cat, baseline values for HR, RR, and SAP were considered as those recorded 5 min before BK. The desired surgical anesthetic depth aimed to maintain the absence of eyelid reflex, ventro-medial globe position, relaxed jaw, absence of spontaneous movement, and exaggerated adrenergic response.

To verify the possible isoflurane sparing effect of the opioids used in premedication, the etIso was first set at 1.5% and modified to ensure the desired surgical anesthetic depth while limiting the autonomic system control response. If a 20% increase in HR and/or SAP and/or RR from what was detected at baseline occured, the etIso would be increased by 0.1%, and 1 µg/kg fentanyl (Fentadon, Dechra Veterinary Products Srl, Torino, Italy) would be administered IV. The total amount of fentanyl required during the procedure was recorded for each cat. In the event of spontaneous movement, the anesthetic depth would also be restored by administering propofol and the total amount was recorded. On the other hand, the etIso would be decreased by 0.1% if the cat showed signs of excessive anesthetic depth, i.e., ≥20% decreased HR and/or blood pressure and/or RR compared to the baseline without showing an autonomous response after surgical stimulation. If marked hypotension (MAP <60 or SAP <100 mmHg) appeared, the anesthetist would first administer a fluid bolus of 2 mL/kg, if hypotension persisted, dobutamine (Dobutamina; Bioindustria L.I.M. Fresonara, Italy) 10 µg/kg/h or ephedrine (Efedrina cloridrato Galenica Senese; Galenica Senese, Monteroni d’Arbia, Italy) 50 µg/kg would be provided IV, according to the anesthetist’s discretion. Any complication was recorded for each cat (hemorrhage, arrhythmia, hypothermia if T < 36.5 °C, etc.).

### 2.3. Post-Operative Assessment

At the end of surgery, isoflurane was discontinued, defining the end of anesthesia (EndA), and cats were disconnected from the breathing circuit. Recovery times were recorded and included the following: regaining of palpebral and swallowing reflexes following extubation and the time of placement in the cage. The observer would also record any particular behavior considered as a ‘secondary effect’, such as opisthotonos; dysphoric signs (pedalage, muscle spasm…); the times for the following: reactions to noise (with eyes opening, looking at the operator, lifting the head), reactions to the operator/owner approach, a search for or avoidance of the operator/owner’s contact, occurrence of urination, defecation, myosis/mydriasis, sneezing, coughing, vomiting, hypersalivation, vocalization, purring; the time of alertness; the time of achieving sternal or sitting positions; the time of attempts to lick the abdomen; and the time of standing. According to the anesthetist’s perception, propofol was administered to smoothen recovery and limit dysphoria or hyper-reactive responses to sounds or touch. Dexmedetomidine was antagonized only if by 1 h from the EndA, the cat still appeared very sedated (i.e., persistence of lateral recumbency, poor responsiveness to stimulation) making pain assessment difficult. Eventual requirement for any drug during the recovery phase was noted, and the overall quality was scored from 1 to 4 (excellent to poor) according to a composite simple descriptive scale (Table 1) modified from an existing scoring system [23].

For the post-operative phase, cats were further divided into two groups differing in the resting regimen according to their living condition: stray cats (group Hosp) were kept in the hospital kennels while domestic cats went home (group Home) the same day of surgery. The English version of the UNESP-Botucatu scale [24] was applied to score pain from 1 h after the EndA and once a day (in the morning) every day for 5 days post op. Owners were trained by our staff on how to fill the UNESP-Botucatu feline pain scale, through a short demo and providing them with a specific link to assess their ability [25]. Buprenorphine 10 µg/kg IM would be administered if a score >3 on Subscale 1 (regarding the psychomotor attitude) or >2 on Subscale 2 (regarding behavior) resulted.

### 2.4. Statistical Analysis

Data were analyzed using the software JASP Team Version 0.18.3 (Amsterdam University, Amsterdam, The Netherlands). Continuous and ordinal data are reported as mean and standard deviation or as median and range, as appropriate. Categorical variables are presented as frequencies and percentages.

Descriptive statistics included age, weight, BCS, living condition (domestic vs. stray), ASA status, opioid group (B, M, P), qualitative and quantitative variables related to behavior, and quality of sedation (visitable patient—yes vs. no); scores for temperament, overall sedation, times for lateral recumbency and for manual approach, presence (yes vs. no), time of occurrence for post-sedation secondary effects (hypersalivation, opisthotonos, dysphoria), and requirement for dexmedetomidine, propofol dose at induction were analyzed.

For the intra-operative phase, the following parameters were considered: requirement (yes vs. no) and total dose of fentanyl as rescue analgesia, anesthesia and surgery time, occurrence of intraoperative complications (hypotension, hemorrhage, hypothermia, hypoventilation, arrhythmia), and times and values of the main intraoperative parameters (etIso, etCO_2_, HR, RR, PAS, T) at the predefined time-points.

Results of the post-operative phase were compared and included recovery score, eventual requirement for propofol, occurrence (yes vs. no) of specific behaviors defined as ‘secondary effects’ during the recovery phase, and times for observing signs of consciousness recovering (i.e., lift head, search for physical contact with the observer, any side effects as opisthotonos, limbs hyperextension, pedalage and myoclonus, hypersalivation, and dysphoria). A further comparison included the resting regimen (Home vs. Hosp) through the application of the UNESP-Botucatu scale from 5 different days (D0–D5), including the eventual administration (yes vs. no) of buprenorphine as rescue analgesia.

Normality was checked with a Shapiro–Wilk test. The equality of variances for continuous variables was analyzed with a Levene test. Differences between the three main groups were tested with ANOVA or a Friedman test, as appropriate; for the post-operative assessment including the Home and Hosp groups, Chi square, Student’s t or Mann–Whitney tests were used, as appropriate. A residual analysis was applied to identify the origin of the difference. A post-hoc analysis was applied using Tuckey’s or Dunn’s tests for multiple comparisons, as appropriate. A univariate logistic regression analysis was performed with intra-operative rescue analgesia (yes vs. no), post-operative rescue analgesia (yes vs. no), and recovery quality score (1–2 vs. 3–4) as dependent variables and age, weight, visitable (yes vs. no), BCS, living condition (stray vs. domestic), temperament score, overall sedation score, opioid, dose of propofol for induction, requirement (yes vs. no) for dexmedetomidine, intraoperative drugs, and fentanyl and number of doses, intraoperative complications, T at the end of anesthesia, recovery score, anesthesia time, surgery time, and presence (yes vs. no) of ‘secondary effects’ as independent variables. A collinearity analysis between variables was performed using the calculated phi coefficients or Cramer’s V coefficients, as appropriate. A *p* value > 0.5 indicated highly correlated variables. Only a limited number of highly correlated variables were included in the models based on the most useful from a clinical perspective. Variables with *p* < 0.1 were included in multivariable logistic regression models. The variables were retained in the model by gradually eliminating non-significant variables backwards. Variables were retained in the model if they significantly reduced the residual deviance of the model. AUC, sensitivity, specificity, and McFadden R2 values were calculated. A McFadden R2 value > 0.2 indicated good model fit. The significance level was set at *p* < 0.05.

## 3. Results

A total of 71 female cats of different breeds were included in the study (group M, n = 25; group B, n = 24; group P, n= 22). Forty were domestic cats, and 31 were stray cats. No significant differences were observed in terms of breed, age, body weight, and BCS. During surgery, 13 cats were pregnant and therefore were excluded from intra- and post-operative statistical analyses (domestic, n = 4; stray, n = 9; group M = 7, group B = 4, group P = 2). Details of the demographic characteristics, scores for temperament, and overall sedation as well as occurrence of side effects, drugs requirement, and onset times are reported in Table 2.

Intra-operative complications were recorded in all groups and are detailed in Table 3, together with physiological parameters that were kept within physiological ranges.

**Figure 1:**
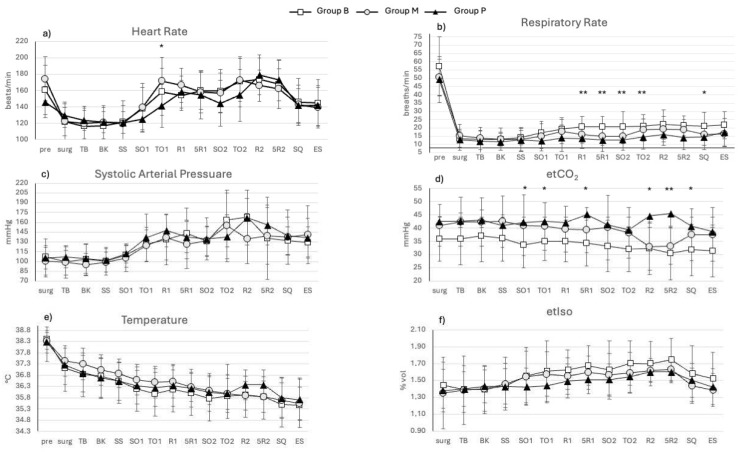
Physiological parameters recorded during anesthesia at predefined time-points in 58 cats undergoing ovariectomy and allocated to 3 different groups: B (butorphanol, white square, n = 20), M (methadone, gray circle, n = 18), and P (pethidine, black triangle, n = 20). (**a**) heart rate; (**b**) respiratory rate; (**c**) systolic blood pressure; (**d**) etCO_2_: end tidal carbon dioxide; (**e**) body temperature; (**f**) etIso: end tidal isoflurane. Pre: pre-sedation; Surg: once moved in surgery room; BT: basal time; BK: Backhaus application; SS: start of surgery; SO1 search for first ovary; TO1: traction of first ovary; R1 first rescue analgesia and 5 min later (5R1); SO2: search for second ovary; TO2: traction of second ovary; R2: eventual second rescue analgesia and 5 min later (5R2); SQ: start of subcutaneous suture; ES: end of surgery (considered as the last skin stitch). Significant differences between groups are reported as * = *p* < 0.05; ** = *p* < 0.01.

No differences resulted in anesthesia (96 ± 28, 98 ± 22, and 94 ± 29 min) and surgery (61 ± 21, 60 ± 17, and 55 ± 15 min) times in group M, B, and P, respectively, whereas physiological parameters showed some differences among groups at some predefined time-points, as reported in Figure 1. The isoflurane requirement ranges between 1.40 and 1.63% in group M, 1.39 and 1.75% in group B, and 1.40 and 1.60% in group P (Figure 1f).

**Figure 2:**
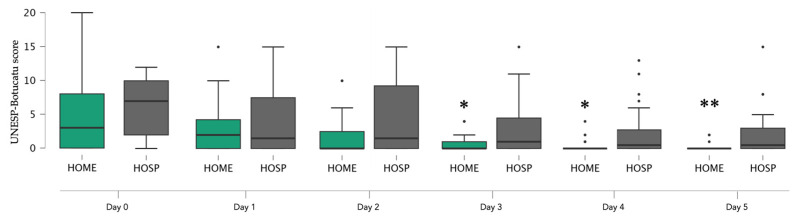
Box plot representing the pain score of the UNESP-Botucatu scale for assessing post-operative pain in cats in Home and Hosp groups. Mann–Whitney test, dots (•) indicate outliers, Day 3* (*p* = 0.041), Day 4* (*p* = 0.004), Day 5** (*p* < 0.001).

Results for the post-operative phase are reported in Table 4. As far as post-operative assessment according to the UNESP-Botucatu scale goes, there was no difference in the incidence of rescue analgesia among groups M, B, and P, and none of the cats required rescue analgesia in the post-operative days from 1 to 5. However, when considering cats based on different rest regimens, there was a significant difference (Table 5, Figure 2).

### 3.1. Factors Associated with Intra-Operative Fentanyl Administration

A univariable logistic regression analysis revealed a number of associated variables that were carried forward for multivariable modeling including the ‘overall sedation score’, ‘dysphoria’, ‘opisthotonos’, ‘dexmedetomidine requirement’, ‘presence of intraoperative complications’, and ‘hypoventilation’. From the result of the final multivariable model (Table 6) the probability of requiring fentanyl intra-operatively was 28 times higher in cats with an overall sedation score of 1 (excellent), and 0.018 times lower in cats scored as 4 (inadequate).

### 3.2. Factors Associated with the Quality of Recovery

A univariable logistic regression analysis revealed a number of associated variables that were carried forward for multivariable modeling including ‘living condition’, ‘occurrence of intra-operative complications’, and ‘hypotension’. The final multivariable model (Table 7) retained domestic cats with a 0.164 times lower risk of a poor recovery score (3–4) compared to stray cats.

### 3.3. Factors Associated with Post-Operative Buprenorphine Administration

A univariable logistic regression analysis revealed a number of associated variables that were carried forward for multivariable modeling including ‘hypotension’, administration of ‘two fentanyl boluses’, ‘recovery score’, and ‘muscle twitching’. From the result of the final multivariable model (Table 8), the probability of receiving post-operative buprenorphine was 0.10 times lower in cats experiencing intra-operative hypotension, 4.85 times higher in cats that received two boluses of fentanyl as rescue analgesia, 0.039 times lower in cats scored as 1 (excellent) at recovery, and 20.03 times higher in cats scored as 2 (moderate); moreover, the probability of a buprenorphine requirement was 12.86 times higher in cats showing muscle twitching at recovery.

## 4. Discussion

This study aimed to compare three anesthetic protocols for cats undergoing elective gonadectomy, evaluating the anesthetic stability and quality of analgesia during and after surgery. The anesthetic protocols included alfaxalone (3 mg/kg) in combination with either methadone (0.3 mg/kg), butorphanol (0.3 mg/kg), or pethidine (5 mg/kg) administered IM in the supraspinatus muscle, which resulted in a superior effect when compared to the quadriceps muscle in terms of quality of sedation and onset time [21]. Regardless of the opioid, cats achieved lateral recumbency within 5 min from the IM injection, but 10 out of the 71 cats (three in group M, two in group B, and five in group P) required IM dexmedetomidine to achieve the desired sedation. Additionally, four out of the five cats in group P required twice the defined dose of dexmedetomidine 5 min apart. This result differs from the findings of our previous study where only three cats receiving butorphanol required dexmedetomidine for persisting inadequate sedation 15 min after premedication [21]. This outcome could arise from the diverse clinical conditions (i.e., ASA status, gender, age…) and requirements for chemical immobilization in the cats of Giannettoni’s study, while in the current study only healthy female cats scheduled for elective ovariectomy where included [21]. Moreover, one of these four cats in group P was not visitable (temperament score of 5), and one had a BCS of 2 that had been associated with a lower level of sedation compared to those with BCS ≥ 3 [21]. Nevertheless, in a study intended to test the analgesic effect of IM pethidine in cats, an obese animal showed severe excitement, dysphoria, and anxiety as obesity is known to increase the volume of distribution for lipophilic drugs into tissues that are not metabolically active [26]. Additionally, a mild level of sedation has been described in healthy cats administered an IM combination of pethidine and midazolam that peaked at 2-5 min and became minimal thereafter; moreover, the same authors reported a highly variable level of sedation among cats in the same group [27]. Finally, the pethidine manufacturer states that if only part of the contents of an ampoule is used, the remaining solution should be kept at room temperature and protected from light if not discarded. As we were storing the unused content in a labelled syringe that was kept in the safe, we cannot guarantee the complete protection from light; therefore, it is likely that some of our cats were experiencing a less optimal level of sedation because they received the content from the syringe rather than from the original ampoule.

Intra-operative physiological parameters were kept within biological ranges in all cats; however, differences appeared among groups at the defined time-points and included etCO_2_, with significantly higher values in group P than B; accordingly, RR was significantly lower in group P than in group B. These differences were also significant at specific time-points and likely dependent on the most painful surgical manipulation, such as the search for the ovary within the abdominal cavity and pedicle traction. This maneuver, most of the time, required the administration of fentanyl as rescue analgesia due to an increase in RR over the predefined cut-off, as per our protocol’s rules, in a greater number of cats of groups B and M, compared to P. Therefore, the greater hyperventilation recorded in groups B and M should justify the difference and the lower etCO_2_ compared to cats in group P at the specific time-points. A similar trend of hyperventilation, in terms of higher RR and lower etCO_2_, was found at the time of subcutaneous closure, which is normally not recognized as a painful stimulation unlike the skin closure [28]; however, it could be due to the too rapid decrease in anesthetic depth toward the end of the procedure, which relied upon the anesthetist’s decision. Furthermore, the higher requirement for pre-operative dexmedetomidine in group P may have accounted for the greater ventilatory depression in these cats that could further worsen the respiratory depressant effect of isoflurane [29]. We should also consider the higher dose of propofol administered at induction in cats of group P, for the lower level of sedation, and this may have contributed to a dose-dependent depression of ventilation and post-induction apnea [30], and the concurrent administration of opioids may have enhanced its effect on ventilation [31].

Heart rate differed significantly only at the time of first ovary traction, with higher values in group M than P. This was not expected as methadone, being a full µ-agonist, was supposed to produce a greater analgesic effect of longer duration than pethidine [32]; undoubtedly, the addition of a multimodal analgesic approach, such as loco-regional anesthesia of the ovarian pedicle [33,34] or at the intraperitoneal and/or incisional level [35], would have likely improved the overall analgesia, but this was not included in our protocol as we meant to evaluate the benefits coming only from the opioids administered in premedication.

No differences resulted on intra-operative rescue administration; however, fentanyl was required in 65, 83, and 90% of cats of group P, M, and B, respectively, and cats in group B required more than one bolus during surgery. This was an expected outcome as butorphanol, although it seems more effective against visceral pain than for somatic pain [36], being a κ-agonist and μ-antagonist opioid, it possesses a lower analgesic effect and a shorter duration than full µ-agonist opioids [16]. Furthermore, butorphanol appears to reach peak blood levels at 20 min after IM administration in cats [37]. Logistic regression in our study revealed a significant correlation between the excellent overall sedation score and the higher intra-operative need for rescue analgesia in cats of group B, confirming the previous considerations of butorphanol’s short duration of action.

Complications were recorded in several cats and included hypothermia in more than 70% of cats in each group, but this is a rather expected complication of general anesthesia in cats due to central nervous system depression, and a source of heating is always highly advocated to limit its occurrence [38]. Arrhythmia was recorded only in two cats, one for each of group B and P and consisted in ventricular premature contractions that did not occur at specific time-points and were self-limiting, resolving without drug administration.

A significant difference was recorded in the occurrence of hemorrhages, which were greater in group M than either B and P. Hemorrhages were considered as bleeding of the ovarian pedicle due to vessel laceration during strumming of the suspensory ligament while handling and manipulating the pedicle. Despite the greater incidence in cats of group M, we do not consider the opioid an influencing cause for the bleeding, but some predisposing factors cannot be excluded as important triggers. For instance, the estrus or diestrus phases of the cycle of some cats in group M, which imply increased ovary blood flow, or the presence of pre-pubertal cats (despite that they were at least 6 months old), which leads to reduced dimensions of the genital apparatus and consequently a greater fragility at surgical manipulation, may be important triggers. However, the bleeding was never quantified, but the entity never required interventions nor specific pharmacological support.

As far as recovery quality and the post-operative phase, cats in group M had a shorter time for lifting the head in response to noise, meaning a quicker recovery of consciousness in about 15 min from isoflurane disconnection compared to more than 20 min in both groups B and P. This faster recovery could depend on the lower requirement for dexmedetomidine in cats of group M, since only one cat received it and was antagonized 1 h from the end of anesthesia; on the other hand, only one cat out of the two in group B that received dexmedetomidine was antagonized 1 h after anesthesia and only two out of four cats in group P received atipamezole. The recovery quality, when broadly grouped as good (scores 1–2) or poor (scores 3–4), showed a lower probability of assigning the worst quality in domestic cats and this is not surprising [39,40].

As far as buprenorphine requirements in the post-operative phase, according to pain assessed with the UNESP-Botucatu, animals with an excellent recovery quality had a lower risk of requiring analgesia, at the same time, when twitching at recovery was recorded there was a greater risk for buprenorphine administration that was also superior in animals that had received two fentanyl boluses during surgery. This is likely because the analgesic plan in these patients was not adequate even at the end of anesthesia to the point of requiring additional rescue analgesia.

In general, according to the UNESP-Botucatu scale cats had similar results, regardless of the opioid administered. However, when considering cats based on different rest regimens, significant differences emerged, showing that cats with home rest experienced lower environmental stress, which was more evident in cats with cage rest at the hospital. This is likely due to the presence of an unfamiliar setting and the absence of a cat-friendly environment [41], which could alter the overall rating scale in non-domestic animals kept in a cage. In fact, an animal at home will necessarily appear more comfortable than an animal confined in a hospital cage; therefore, it may show fewer signs of stress and discomfort, when scored with the UNESP-Botucatu scale. Several animals had a feral behavior if they were a stray cat, and it is likely that the clinical assessments, from the time of recovery until the subsequent post-operative phases, were somehow altered by the presence of the observer and not necessarily linked to the presence of pain. In fact, by nature, they are not used to the presence of an observer and therefore they may be afraid of them; moreover, it has been reported that demeanor can contribute to a misclassification of pain in cats [42]. Cats are unique and likely alter their behavior, especially when fear, anxiety, and stress are present in a hospital setting. Signs of pain may be subtle and misinterpreted such as when a cat simply reduces movement and ‘freezes’ due to fear. Moreover, caregivers and owners can be trained as well to improve their pain assessment skills and several websites are available for this purpose [8,42].

The rationale for choosing to administer rescue analgesia according to the cut-off calculated for the single subscales 1 and 2, rather than for the total score, relied upon the idea of limiting possible hidden or overexpressed items given the presence of stray animals. Moreover, these subscales showed the same excellent properties as the scale total score for global pain assessment [24].

Having said that, the three proposed protocols could represent a valid alternative, especially in the most critical patients (i.e., ASA > 2) where metabolization of induction anesthetics, such as cyclohexamines, may be compromised [43]. In the study of Giannettoni and collaborators [21] the enrolled population was not homogeneous for age and gender, nor was it standardized by pathology or reason for chemical restraint, but, in particular in cats with ASA > 1, a good quality of sedation and maintenance of cardiorespiratory parameters within physiological ranges were recorded as well as optimal quality of recovery.

The use of alfaxalone has been largely described for many surgical procedures in the feline species [15,18,19,20], although this does not mean that the protocols were equivalent. In fact, from our results, some critical issues have emerged. First of all, if sedation is less than optimal, stress for the cat increases and, consequently, the duration of pre-surgical preparation. Regardless of the opioid, in every group, an average of 30 min from sedation was required to enter the operating room. Among the factors that affected this extended time for cat preparation, we consider primarily the occurrence of opisthotonos and dysphoria, frequently in combination with alfaxalone administration, that made the physical approach for intravenous catheterization rather difficult. Prolonging the patient’s preparation can lead to an exhaustion of the analgesic effect of the opioid administered in premedication during the surgical procedure and, therefore, inadequate analgesic activity for the most painful surgical phases [44]. In order to limit this outcome, we recommend, especially in healthy and more feral cats, to support the premedication with the addition of a low dose dexmedetomidine (i.e., 3 µg/kg IM).

Some limitations are worth mentioning for this study. First, a higher number of animals with a better distribution among groups, especially regarding living conditions, is advocated to lower the bias of selection in our results. Second, the inclusion of more animals categorized with similar age and BCS may be warranted as well. The presence of stray animals with the tendency of feral behavior may have made a proper pain assessment difficult for the observer. Finally, as this study was carried out in a teaching environment, the presence of students, who are not necessarily experts in surgery and anesthesia activities, may have contributed to producing different times and management.

## 5. Conclusions

In conclusion, this study highlights that premedication with alfaxalone combined with the three investigated opioids did not cause any clinically relevant cardiovascular or respiratory alterations in healthy cats maintained in isoflurane and undergoing elective OV.

As far as the choice of the opioid, we highly suggest considering animal behavior. In fact, pethidine has demonstrated an overall greater cardiorespiratory stability than the other two opioids, but a lower quality of sedation, discouraging its combination with alfaxalone in more feral cats, unless coupled with dexmedetomidine.

Furthermore, from the perspective of a multimodal anesthetic approach, it is highly recommended that there is a combination with a locoregional analgesia at the ovarian pedicle before manipulations, so as to increase intra- and post-operative analgesic management and to reduce the administration of opioids in response to the most painful surgical events.

## Figures and Tables

**Table 1:** Simple descriptive scale used to assess recovery from general anesthesia, modified from Khenissi et al., 2017 [23].

Score	Description
1-Excellent (no struggling)	No signs of residual effects and no hyper-reactive response to sound or manual assistance.
2-Moderate (mild struggling)	Some assistance to stand, minimal residual effects, and minimal to no hyper-reactive response to sound or manipulation.
3-Fair (moderate struggling)	Repeated attempt to stand, requires assistance, unstable, moderate hyper-reactive response to sound and/or manipulation, residual effects of anesthetics.
4-Poor (prolonged struggling)	Unable to stand without assistance, hyperkinesis in response to manual assistance, response to sound, clear residual effects of anesthetic.

**Table 2:** Demographic characteristics, scores, and times related to sedation and incidence of side effects reported in 71 cats undergoing surgery.

Parameter	Group M (n = 25)	Group B (n = 24)	Group P (n = 22)
Age (months)	12 ± 8	14 ± 11	13 ± 11
Weight (kg)	3 ± 0.6	3.1 ± 0.4	3 ± 0.3
BCS [1–5]	3 [1–4]	3 [2–4]	3 [2–4]
At admission: Visitable (n°)	19	14	14
Not visitable (n°)	6	10	8
Temperament score [1–5]	2 [1–4]	2 [1–5]	1.5 [1–5]
Overall sedation score [1–4]	2 [1–4]	1 [1–4]	2 [1–4]
Incidence of: opisthotonos (n°)	6	3	5
hypersalivation (n°)	11	6	8
dysphoria (n°)	8	2	8
Requirement for dexmedetomidine (n°)	3	2	5
Total of dexmedetomidine doses (n°)	3	2	9 *
Time for lateral recumbency (min from T0)	3.8 ± 1.7	2.9 ± 0.8	3.0 ± 0.8
Time for manual approach (min from T0)	7.8 ± 6.5	6.9 ± 6.6	11.1 ± 13.3
Time for induction (min from T0)	19.8 ± 8	17.8 ± 6.4	25.6 ± 15.7
Time for entering the theatre (min from T0)	28.8 ± 8.3	26.8 ± 8.6	35.4 ± 16.5 ^§^
Propofol induction dose (mg/kg)	3.3 ± 1.6	3.2 ± 1.4	3.8 ± 2.2

Results are reported as mean ± SD, median [range] for continuous variables, number of animals for categorical variables. n° = number of cases; BCS = body condition score; T0 = time of IM injection of the anesthetic mixture that included alfaxalone and one among 3 opioids: methadone (M), butorphanol (B), pethidine (P). * *p* = 0.021; ^§^ *p* = 0.03.

**Table 3:** Physiological parameters recorded before the procedure (pre) and at basal time during anesthesia; occurrence of intra-operative complications and requirements for additional drugs in 58 cats undergoing ovariectomy allocated to 3 groups according to the opioid administered IM with alfaxalone: methadone (M), butorphanol (B), pethidine (P).

Parameter	Group M (n = 18)	Group B (n = 20)	Group P (n = 20)
HR (beats/min) pre basal	174 ± 27 120 ± 19	161 ± 30 111 ± 34	146 ± 19 124 ± 12
RR (breaths/min) pre basal	51 ± 11 14 ± 7	57 ± 18 13 ± 5	49 ± 14 12 ± 7
T (°C) pre basal	38.3 ± 0.4 37.3 ± 0.7	38.4 ± 0.5 36.8 ± 0.8	38.3 ± 0.5 36.9 ± 0.9
SAP (mmHg) basal	98 ± 19	100 ± 25	107 ± 16
MAP (mmHg) basal	62 ± 15	68 ± 15	71 ± 15
DAP (mmHg) basal	42 ± 14	47 ± 15	50 ± 14
Complications: Hypotension (n°)	6	6	5
Arrhythmia (n°)	0	1	1
Hypoventilation (n°)	0	1	1
Hemorrhage (n°)	6 *	2	1
Hypothermia (n°)	15	15	14
Fentanyl rescue (n° R1–R2)	15–12	18–16	13–8
total µg/kg	1.8 ± 0.6	1.9 ± 0.8	1.6 ± 0.6
Requirements for: Propofol (n°)	4	4	1
mg/kg	1.7 ± 1.5	1.3 ± 0.6	1
Dobutamine (n°)	0	1	0
µg/kg/min	0	7.7	0
Ephedrine 50 μg/kg (n°)	1	2	0

Results are reported as mean ± SD and number. HR = heart rate; RR = respiratory rate; T = temperature; SAP = systolic arterial pressure; MAP = mean arterial pressure; DAP = diastolic arterial pressure; R1 = first rescue; R2 = second rescue; pre = values recorded before drugs administration; basal = values recorded 5 min before Backhaus application; * *p* = 0.039.

**Table 4:** Results of physiological parameters and side effects recorded at recovery in 58 cats undergoing ovariectomy according to the groups: methadone (M), butorphanol (B), pethidine (P).

Parameter	Group M (n = 18)	Group B (n = 20)	Group P (n = 20)
HR at EndA (beats/min)	162 ± 43	147 ± 32	142 ± 28
RR at EndA (breaths/min)	22 ± 11	22 ± 9	18 ± 8
SpO_2_ at EndA (%)	98 ± 2	99 ± 2	97 ± 6
T at EndA (C°)	35.9 ± 0.9	35.6 ± 1.1	35.8 ± 0.9
Palpebral reflex from EndA (min)	2 ± 2	1.5 ± 1	2 ± 2
Extubation from EndA (min)	2.5 [1–10]	2 [1–8]	3.5 [1–12]
Transfer to cage from EndA (min)	5 [2–32]	4 [2–13]	6 [1–13]
Side effects: Opisthotonos (n°)	7	8	7
Limb hyperextension (n°)	7	9	9
Pedalage and myoclonus (n°)	5	7	4
Hypersalivation (n°)	11	14	13
Dysphoria (n°)	3	5	3
Drugs at recovery: Propofol (n°, mg/kg) Atipamezole (n°, µg/kg)	2, 2.5 ± 2 1, 4	2, 2.7 ± 1 1, 8	1, 1 2, 5 ± 2
Lift head (min from EndA)	15 ± 6.5	22 ± 9.5	27 ± 14 *
Search for operator contact (min from EndA)	21 [9–25]	34 [16–57] ^§^	31 [23–62]
Recovery quality (score 1–4)	1 [1–3]	1 [1–3]	1 [1–4]
Requirement for buprenorphine rescue (n°)	7	8	6
Time to buprenorphine rescue (min from EndA)	134 ± 19	113 ± 17	116 ± 40
UNESP-Botucatu (total score)			
Day 0	3.5 [0–20]	4 [0–20]	4 [0–12]
Day 1	1.5 [0–15]	2.5 [0–15]	1.5 [0–11]
Day 2	1 [0–10]	0 [0–15]	0 [0–12]
Day 3	0 [0–11]	0 [0–15]	0 [0–10]
Day 4	0 [0–7]	0 [0–13]	0 [0–1]
Day 5	0 [0–5]	0 [0–15]	0 [0–8]

Results are reported as means ± SD or median [range] for continuous variables, and as number for categorical variables. HR = heart rate; RR = respiratory rate; T = temperature; SpO_2_ = hemoglobin oxygen saturation; EndA = end of anesthesia. * *p* = 0.008; ^§^ *p* = 0.024.

**Table 5:** Results of post-operative data in 58 cats undergoing ovariectomy and divided according to the resting regimen: at home (group Home) or confined in a cage (group Hosp).

Parameter	Home (n = 36)	Hosp (n = 22)
Recovery quality (score 1–4)	1.3 [1–3]	1.8 [1–4]
Requirement for buprenorphine rescue (n°)	12	9
Time to buprenorphine rescue (min from EndA)	130 ± 20	108 ± 30
UNESP-Botucatu (total score)		
Day 0	3 [0–20]	7 [0–12]
Day 1	2 [0–15]	1.5 [0–15]
Day 2	0 [0–10]	1.5 [0–15]
Day 3	0 [0–4] *	1 [0–15]
Day 4	0 [0–4] *	0.5 [0–13]
Day 5	0 [0–2] **	0.5 [0–15]

Results are reported as means ± SD or median [range] for continuous variables, and as number for categorical variables. EndA = end of anesthesia. * *p* < 0.05; ** *p* < 0.001.

**Table 6:** Multivariable model for risk factors associated with intra-operative fentanyl administration as rescue analgesia.

Intraoperative Rescue Analgesia (Fentanyl)	OR	95% CI	*p*-Value
Grade 1 Overall sedation score	Ref		
Yes	28	3.8–205.8	<0.001 **
Grade 2 Overall sedation score	Ref		
yes	0.018	0.002–0.2	0.001 *

Area under the curve = 0.887; Sensitivity = 0.935; Specificity = 0.667; R^2^ McFadden = 0.41; OR = Odds ratio; CI = Confidence interval; Statistical level set at *p* < 0.05; * *p* < 0.05; ** *p* < 0.001.

**Table 7:** Multivariable model for risk factors associated with the quality of recovery.

Recovery Quality Score	OR	95% CI	*p*-Value
Cat’s Living condition	Ref		
Domestic cats	0.164	0.028–0.952	0.044 *

Area under the curve = 0.893; Sensitivity = 0.444; Specificity = 0.980; R^2^ McFadden = 0.39; OR = odds ratio; CI = Confidence interval; * Statistical level set at *p* < 0.05.

**Table 8:** Multivariable model for risk factors associated with post-operative buprenorphine administration.

Post-Operative Rescue Analgesia (Buprenorphine)	OR	95% CI	*p*-Value
Grade 1 Overall sedation score	Ref		
Yes	0.039	0.004–0.413	0.007 *
Grade 2 Overall sedation score	Ref		
Yes	20.53	2.2–192.5	0.008 *
Muscle twitching	Ref		
Yes	12.86	1.5–110.3	0.02 *
Hypotension	Ref		
Yes	0.10	0.014–0.8	0.03 *
Two doses of fentanyl	Ref		
Yes	4.85	1.12–21.1	0.034 *

Area under the curve = 0.828; Sensitivity = 0.762; Specificity = 0.865; R^2^ McFadden = 0.4; OR = Odds ratio; CI = Confidence interval; * Statistical level set at *p* < 0.05.

## Data Availability

The datasets used and/or analyzed during the current study are available from the corresponding author on reasonable request.
